# Adaptation and psychometric investigation of the Gameful Experience Questionnaire (GAMEFULQUEST) in Brazilian Portuguese

**DOI:** 10.1038/s41598-024-68101-7

**Published:** 2024-07-26

**Authors:** Luiz Oliveira da Silva Junior, Wilk Oliveira, Juho Hamari

**Affiliations:** 1 Federal University of Paraíba, Rio Tinto, Brazil; 2 Gamification Group, Faculty of Information Technology and Communication Sciences, Tampere University, Tampere, Finland

**Keywords:** Human behaviour, Statistics

## Abstract

Over the years, the use of questionnaires has become one of the most used methods for analyzing individuals’ experiences. Especially in the area of gameful environments (e.g., games, gamification, and simulators), the Gameful Experience Questionnaire, a self-report instrument to measure gameful experience, became one of the most popular. Despite the instrument’s popularity, there is no Brazilian Portuguese version, preventing studies from being carried out in Brazil (i.e., a country with more than 200 million inhabitants), where only 5.1% of the population have adequate English comprehension skills. To face this challenge, we conducted a cross-cultural adaptation of the Gameful Experience Questionnaire, providing a version of the questionnaire in the Brazilian Portuguese language. For this process, we conducted a mixed-methods (i.e., qualitative and quantitative) psychometric study (N = 384) organized in six steps (i.e., (i) translation, (ii) synthesis, (iii) experts evaluation, (iv) target audience evaluation, (v) adapted instrument application, and (vi) validation (i.e., confirmatory factor analysis)). The results indicate that the cross-cultural adaptation took place efficiently, where the resulting instrument maintained the psychometric properties of the original, measuring the construct of interest with similar effectiveness (i.e., $\chi ^2/df$ = 2.4, RMSEA = 0.061, CFI = 0.991, TLI = 0.989, GFI = 0.986 and SRMR = 0.061), enabling its application with Brazilian Portuguese speakers. With this study, we contribute to researchers and practitioners in the field of gameful environments by providing an instrument to measure gameful experience in the Brazilian Portuguese language.

## Introduction

Gameful environments (i.e., environments that encapsulate the subjective perception of users while interacting with such environments, encompassing elements of challenge, autonomy, and meaningfulness^1^), whether in the form of gamified environments (“gamification” is considered the process in which services, activities, and systems are transfigured to promote similar motivational benefits as found in games”^2,3^), simulations, or actual games, is an emerging field in digital design, user engagement studies, and social behavior and interaction^3–5^ and tends to immerse users in an interactive and engaging environment, fostering a sense of enjoyment and accomplishment^6–8^. Thus, understanding and measuring these experiences are vital for optimizing the design and impact of gameful interventions^9–11^.

In the path of understanding the individuals’ experience when using some type of environment, within the realm of psychological research, the utilization of self-report measures instruments holds significance^12–14^. These instruments serve as important tools for capturing individuals’ subjective experiences, attitudes, and perceptions, offering a comprehensive understanding of complex psychological constructs^12,13,15^. Their popularity is based on several persuasive advantages, such as easy interpretability, the richness of information, motivation to report, causal force, and sheer practicality^16^. Particularly within the dynamic landscape of gameful experiences, self-report measures provide a direct means of assessing users’ perceptions and evaluating the effectiveness of gameful interventions, thereby informing the design and implementation of future initiatives^9,17–20^.

In the field of gameful environments, the Gameful Experience Questionnaire (GAMEFULQUEST), an instrument devised by Högberg, Hamari, and Wästlund^1^, stands as a pivotal and popular tool for evaluating users’ gameful experiences within diverse environments^21–23^. Originally developed in English, this questionnaire captures essential facets of gameful engagement (i.e., accomplishment, challenge, competition, guided, immersion, playfulness, and social experience), providing researchers with a reliable and comprehensive means of assessment^1^. **Accomplishment** is experiencing the demand or drive for successful performance, goal achievement, and progress^1^, **Challenge**, in turn, is experiencing demand for a great effort to be successful, thus the ability of the person is tested^1^, **Competition** is related to experiencing rivalry towards one or more actors (self, another person, service, or group) to gain a scarce outcome that is desirable for all actors^1^, while **Guided** means experiencing being guided on how (including what and when) to do, and on how to improve the target behavior^1^. **Immersion** is when all attention is taken over, and the person experiences being absorbed in what he or she is doing while having a sense of being dissociated from the real world^1^, **Playfulness** is defined as the experience of being involved in voluntary and pleasurable behaviors that are driven by imagination or exploration while being free from or being under spontaneously created rules^1^, and **Social experience** are the experiences emanating from the direct or indirect presence of people (both present in the real world and in the service), service-created social actors, and service as a social actor^1^.

Despite being a recent instrument, the GAMEFULQUEST already has consolidated solidity, having been attested in previous works, such as its validation, carried out in the third study conducted by the authors, aiming to demonstrate the efficiency of the instrument, which presented indices extremely positive adjustment parameters, which will be mentioned in our discussion, generating a final version that is efficient in the task of measuring the constructs intended by the instrument. Likewise, Booysen^24^, in which the GAMEFULQUEST was also subjected to an adaptation process, being answered by 308 employees of a retail company, presenting positive fit indices, and proving efficient in measuring users’ gameful experience in a South African gamified online training context. However, the global applicability of such instruments demands cross-cultural adaptation and validation, once we know that for measures to be used across cultures, the items must not only be translated well linguistically but also must be adapted culturally to maintain the content validity of the instrument^25^, ensuring their relevance and reliability in diverse linguistic and cultural contexts^26^.

Especially, in the context of Brazil, a South American country with more than 200 million inhabitants, where English proficiency is not universal (i.e., only 5.1% of the population have adequate English comprehension skills^27^), it is important to validate instruments in Brazilian Portuguese providing opportunities for the use of these instruments in both industry and academia^28^. Thus, advancing the literature, our study addresses this gap by undertaking the cross-cultural adaptation of the GAMEFULQUEST Questionnaire into Brazilian Portuguese, adhering to established guidelines for translation, synthesis, expert evaluation, and statistical validation. By doing so, we aim to contribute to the accessibility and applicability of psychometrically sound instruments in the Brazilian context, facilitating nuanced research on gameful experiences.

To achieve this goal, we employed a systematic methodology (both qualitative and quantitative) psychometric study involving six different steps psychometric study (N = 384) organized in six steps (i.e., (i) translation, (ii) synthesis, (iii) experts evaluation, (iv) target audience evaluation, (v) adapted instrument application, and (vi) validation (i.e., confirmatory factor analysis (CFA))).

Our main results indicate that the model structure is adequate (i.e., $\chi ^2/df$ = 2.4, RMSEA = 0.061, CFI = 0.991, TLI = 0.989, GFI = 0.986 and SRMR = 0.061). Thus, we provide an adapted version of the instrument in Brazilian Portuguese. Furthermore, our study, as far as we know, is the first to execute the transcultural adaptation of the GAMEFULQUEST Questionnaire to the Brazilian Portuguese language. Thus, this study’s contribution lies in bridging the gap between global research trends in gameful environments and the linguistic diversity of the Brazilian population.

## Method

**Figure 1:**
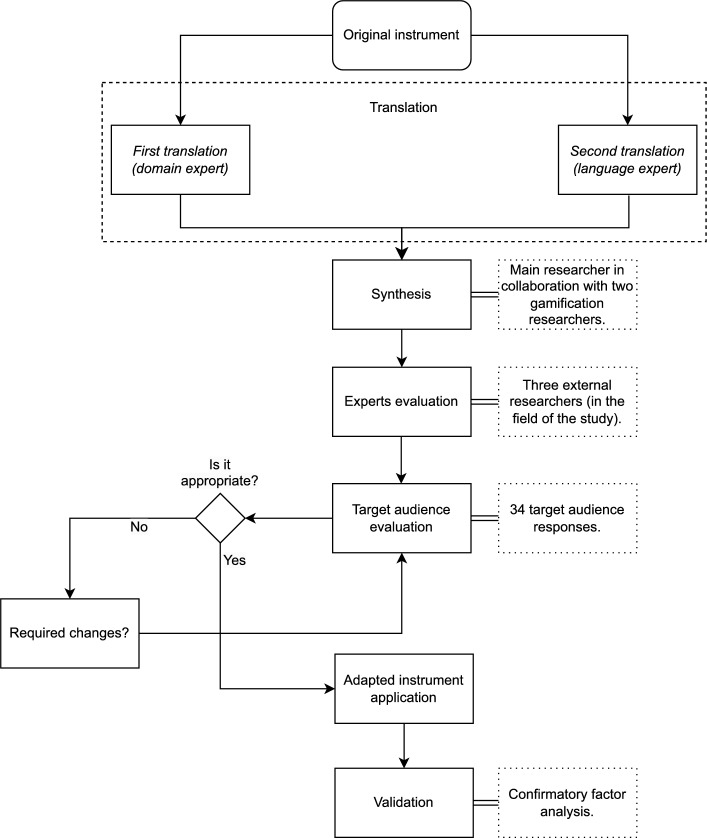
Study’s method.

This study aimed to conduct a cross-cultural adaptation of the GAMEFULQUEST Questionnaire^1^ in Brazilian Portuguese and analyze its psychometric properties. The GAMEFULQUEST is a self-report instrument, proposed by Högberg, Hamari, and Wästlund^1^, originally in English, that aims to measure the users’ gameful experience while using a gameful environment (e.g., a game, gamified system, or a simulator). To ensure that the instrument will maintain its original characteristics, and measure the same factors, it’s necessary to consider the cultural, idiomatic, linguistic, and contextual aspects^29^. Thus, we followed the steps proposed by Borsa, Damásio, and Bandeira^30^, consisting of six steps (i.e., (i) translation, (ii) synthesis, (iii) experts evaluation, (iv) target audience evaluation, (v) adapted instrument application, and (vi) validation (i.e., in our case, based on CFA)) to perform a cross-cultural adaptation of an instrument. Figure 1 presents our study’s method.

The *first step* (i.e., translation), consists of a double translation of the original items, made by two distinct translators that need to be natives in the target language, and fluent in source^25^. That first phase took from April 13th to May 19th, 2023. To keep the adapted items with a good balance between academic language terms and the popular language of the target audience, Borsa, Damásio, and Bandeira^30^ recommend that one of the translators needs to be familiar with the items of the main construct, while the other, preferably, should not be aware of the translation objective. Following the recommendation, we sent the original items (in separate Excel templates), to two contributors (one in the field of gameful environments and another expert in Brazilian Portuguese and English language) and asked them to perform the translation and send back the archive, with the translated items and their considerations.

The *second step* (i.e., synthesis), aims to create a unique version, summarizing the two translated versions generated by the previous step^30^. Borsa, Damásio, and Bandeira^30^ describe that this act compares the different translations and assesses their semantic, idiomatic, conceptual, linguistic, and contextual differences, with the sole purpose of creating a single version. This process should be done by the main researcher with a minimum of two more judges, so that decisions do not become unilateral, and each item needs to be evaluated separately^30^. Following the recommendation, we conducted the synthesis with the main researcher in collaboration with a researcher in the field of gameful environments, discussing item by item in an online meeting through the Google Meet platform. This phase took from May 19th to May 26th, 2023, and generated the first version of the adapted items, which was used in the next phase.

The *third step* (i.e., experts evaluation) consists of the expert’s evaluation of the resultant version of the items after the synthesis by a group of experts in the area of psychological evaluation, or in the main construct of the items translated^30^. These experts should assess aspects related to the structure, layout, instrument instructions, scope, and adequacy of expressions contained in the items after the synthesis^30^. This step becomes further important if the study aims for a population different from the researcher’s conditions, because of the possibility of layout and language mistakes (e.g., a Questionnaire destined for elderly people, adapted by a young student, with a completely different vocabulary and historical context)^30^. After the evaluation, the items can be modified, according to the suggestions of the experts, and, after that, the first version of the adapted instrument is ready to be evaluated by the target audience^30^. Following the recommendations, this step was done by three external researchers (in the field of the study), with extensive knowledge of the construct. We send the items resultants of the second step by email (in separate Excel templates), and they send back with their considerations. As guided by Borsa, Damásio, and Bandeira^30^, and upon receipt of materials, an analysis of the suggestions was executed, and some modifications were accepted, resulting in the second version of the items. This phase lasted from May 27th to July 21st, 2023.

The *fourth step* (i.e., target audience assessment) consists of evaluating the items by a group of subjects with the characteristics of the target audience, and covering a certain level of variation (e.g., if the Questionnaire aims to be answered by elderly people from a whole country, is advisable to have subjects from different regions and with variate ages, but all between 50 and 80 years)^30^. This procedure investigates whether the instructions are clear, whether the terms found in the items are appropriate, whether the expressions correspond to those used by the group, and other aspects^30^. The subjects are encouraged to suggest modifications in the items if they judge necessary, and the objective is to reach the saturation criterion, which is when the suggestions become repeated^30^. At the same time, according to Borsa et al.^30^, the process can be repeated more than one time, depending on the level of modifications realized, and, after all the items are approved, without any new suggestions, the instrument is ready for the next stage, the adapted instrument application. Following the recommendations, we sent invitations to email lists and groups of social media, with the announcement of the second version of the questionnaire, to achieve the minimum answers required to reach the saturation criterion. This action started on August 1st, and the answers continued until August 18th, 2023. After receiving 32 answers, we reached the goal (i.e., saturation), with the suggestions becoming repetitive, reaching an inter-rater agreement above 80%^31^, without adding new changes to be made, which indicated that the items were clear and understandable for the intended audience.

The *fifth step* (i.e., adapted instrument application) consists of the beginning of the Questionnaire validation process^30^. The previously mentioned adaptation processes aim to yield instruments that are equivalent across different cultures^30^. In this step, the items resultants of the previous four steps should be organized in an aleatory order and applied to a proper number of participants, aiming to collect sufficient answers to validate the process of adaptation (statistical analysis). Following the recommendations, we used the strategy of disclosure among email lists, social media, and contact with educational institutions (e.g., universities).

The *sixth step* (i.e., validation) consists of the data analysis of the data obtained at the adapted instrument application^30^. After reaching the required number of answers, all the data obtained should be statically evaluated to ensure that the original objectives are maintained, even after the adaptation^30^ and test validity assesses whether the test measures what it purports to measure^32^. Neither in an adaptation, is necessary to test that point, since the adaptation for another context can change the main meaning of the original items^32^. The steps required during the validation of a psychological instrument are diverse^33^, and the correct order and execution of them, consequently, will create a valid new version. Following the recommendations of Borsa et al.^30^, we performed a series of tests, including internal reliability, to measure whether the internal structure of the instrument remained strong and intact, as well as correlation tests, to observe whether this phenomenon was present among the items of each dimension, as well as with the entire instrument. Finally, the CFA test was performed to measure whether the factorial load of the items remained high, even after being adapted to a new language and context.

### Data gathering

For this study, the adapted instrument was applied as an online survey, using the Google Forms platform [https://docs.google.com/forms/]. Following the original study^1^, the 56 adapted items of the questionnaire were presented on a 7-point Likert scale^34^, with the items separated in the seven dimensions proposed by the original instrument, randomized within each section. Following the recommendations of Kung, Kwok, and Brown^35^, as well as following the example of recent similar studies in this field^10,20,36^, we inserted an “attention-check” item (i.e., “**I feel good, but this is a question to check if you are paying attention to the form. If you read this question, select option 4**.” | “*Me sinto bem, mas, essa é uma pergunta para checar se você está prestando atenção no formulário. Se você leu esta pergunta, marque a alternativa 4*.” (in Brazilian Portuguese)) in the fourth section/dimension, to prevent responses made by inattentive participants from making their way to the final analyses.

The data gathering occurred between August 30, 2023, and February 10, 2024, reaching a total of 411 answers, divided into two Google Forms questionnaires. In the *first one*, we direct responses to a single platform, the Duolingo [https://pt.duolingo.com/] (i.e., a gamified app focused on teaching languages widely used in formal and informal education). We oriented the participants to use the platform for a minimum time of 20 min so that they had a minimum experience capable of providing a basis for answering the questionnaire. In turn, for the *second one*, we advised participants to use a gameful platform of their choice, and we included a field for it to be indicated, in the forms. We decided to provide this type of choice to reach a larger sample, since with the possibility of using only Duolingo, a large enough quantity had not been obtained for the analysis. At the end of the response collection period, in the first form, 261 responses were obtained, of which 16 were invalid (due to a wrong answer in the “attention-check” item), and in the second form, 150 responses were obtained, of which 11 were invalid (due to a wrong answer in the “attention-check” item). The answers were combined into a single dataset, considering that the objective of the study is to analyze the applicability of the questionnaire regardless of the specific type in a gameful environment.

### Participants description

For the *first step* (i.e., translation), the selected participants, following the previous instructions for the trans-cultural adaptation, given by Borsa, Damásio, and Bandeira^30^, were selected based in their knowledge and language skills. For the translation phase, the first translator selected was a gamification researcher, a self-declared male, aged 22 years, with experience with the construct, having published scientific studies, while the second was a lay person, self-declared male, aged 31 years, fluent in the English language, but without specific knowledge of gamification, being an ordinary translator, to keep the language as close to the general population as possible.

In the *second step* (i.e., synthesis), the participants selected encompassed the two first authors of the study, with a collaboration of another gamification researcher, a self-declared female, 32 years, with experience with the construct, and previous experience with the application of scales for measurement of the gamification construct effects.

The *third step* (i.e., experts evaluation), the participants of this phase were three experts in the gamification construct, two self-declared males, with ages of 47 and 25, and a self-declared female, aged 45. Both selected candidates have extensive experience with the construct,

The *fourth step* (i.e., target audience assessment), reached 32 people, ages between 19 and 60 years old, and the most varied levels of knowledge, social class, and occupations, such as university students, workers, postgraduate teachers, psychologists, and retirees. The average age of the participants is 26.7, with a standard deviation of 7.9 and a variance of 63.4.

For the CFA, we obtained 411 total answers, of which 27 were discarded for getting the “attention-check” item wrong. Thus, the final sample size was composed of 384 answers, 152 self-declared as female, 219 self-declared as male, and seven self-declared as non-binaries. Also, six participants chose not to declare their gender. The participants were distributed between 19 states in the country, and the Federal District, covering the five geographic regions of Brazil, with a predominance of the states São Paulo (42%), Paraiba (26%), and Paraná (4.68%). The age group was very diverse, with participants between 15 and 67 years old. The major quantity was of young people, between 15 and 20 years (52%). Two averages were calculated, related to the age of the participants, the average age of all respondents, which was 37, with a standard deviation of 14.03, and a variance of 196.85, and then, the average of responses by age, aiming to identify which specific age groups fit into showed more presence in the sample. This average was 8.93, with a standard deviation of 13.57 and a variance of 184.16. Despite the negative difference in the number of responses achieved, which reached 54% of the value suggested by the calculator A-priori^37^, in recent literature attests that, when the factorial loads of the items reach significant values, the sample size can be reduced, without compromising the validity of the result^38^.

### Statistical analysis

After data gathering, we started the analysis, where we analyzed (i) internal reliability (i.e., (Cronbach’s $\alpha$ and McDonald’s $\omega$)), (ii) correlations, (iii) dimension distribution, and (iv) CFA. Considering that the study aims to confirm the efficiency of the instrument GAMEFULQUEST, according to Levine^39^ a CFA is the most indicated type of analysis, if we compare it with Exploratory Factor Analysis (EFA), since there is already a validated instrument, with a consolidated theoretical structure^40^.

The data were analyzed using IBM SPSS 27^41^ and JASP 0.18.3^42^. The IBM SPSS 27^41^ software was used to conduct a Shapiro-Wilk test^43^ and measure the internal reliability (i.e., Cronbach’s $\alpha$ and McDonald’s $\omega$) in the dataset, to prove the consistency with which the items, even if different from each other, in a single test, measure the same construct, ensuring that it is stable in all its components^44^. In turn, the software JASP 0.18.3^42^ was used to conduct the CFA, using structural equation modeling (SEM), with a robust diagonally weighted least squares, which is the most appropriate for the questionnaire, since it presents the most popular technique for dealing with categorical data^45^, and is stable even with deviation from normality, and samples of varying sizes^46^, which uses a Likert response pattern. It was also measured in the CFA process the factor correlations. Was used the Shapiro-Wilk test^43^, the most powerful test for all types of distribution and sample sizes^47^, to show if our data does not follow a normal distribution, dimension distribution test target. To evaluate the validity of the adapted instrument, we analyzed the model Chi-Square ($\chi ^2$), the Relative Chi-square ($\chi ^2/df$), the Goodness of Fit Index (GFI), the Tucker-Lewis Index (TLI), the Comparative Fit Index (CFI), the Standardized Root Mean Square Residuals (SRMR) and the Root Mean Square Error of Approximation (RMSEA) results. Based on different studies’ recommendations^48–52^ we considered the goodness-of-fit indexes as $\chi ^2$ p $\ge$ 0.05; $\chi ^2/df$ $\le$ 3; GFI $\ge$ 0.95; TLI $\ge$ 0.95; CFI $\ge$ 0.95; NFI $\ge$ 0.95; SRMR $\le$ 0.08; and RMSEA $\le$ 0.06.

### Ethical statements

This study has been performed following the Brazilian National Health Council resolution number 510 published on April 7th, 2016, and with the relevant guidelines and regulations set by the Universities involved. Informed consent for participation was obtained from all participants.

## Results

In this section, we present the results from the analyses of internal reliability, dimension distribution, correlations presented between the dimensions, and the results from the CFA.

### Confirmatory factor analysis

**Figure 2:**
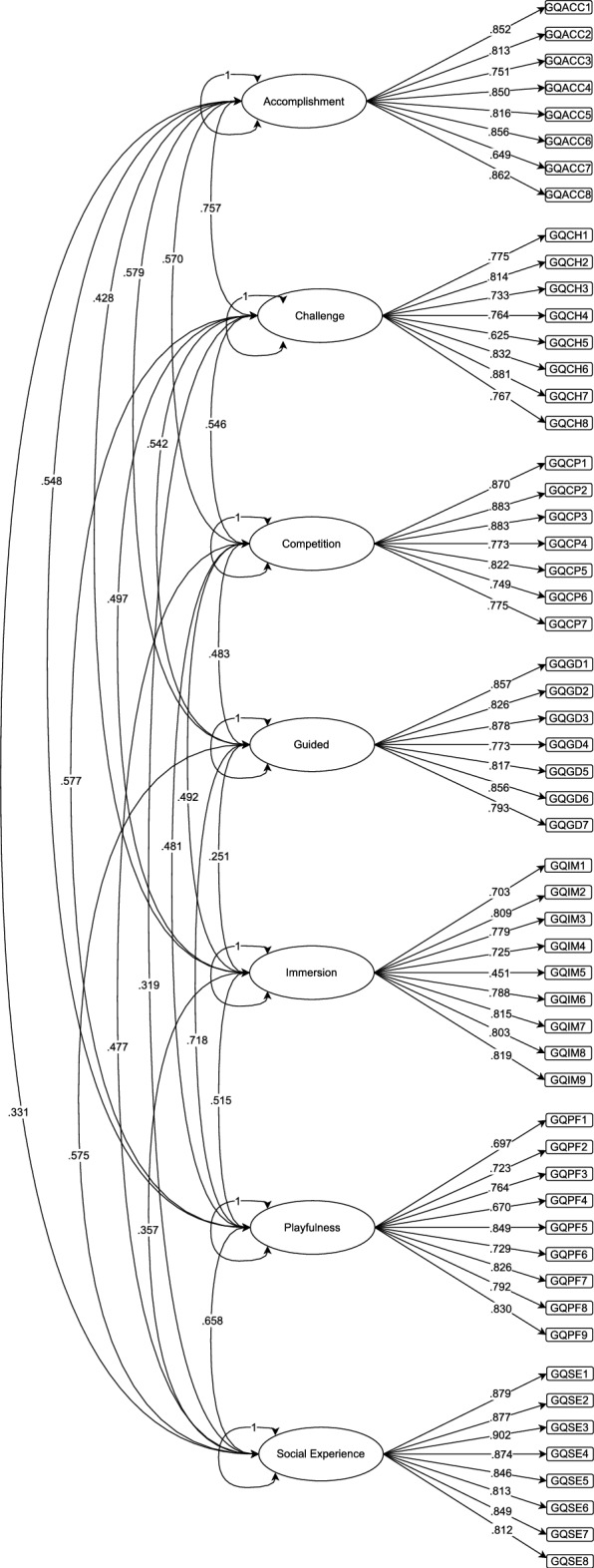
Path model with correlations between the factors. The ellipses represent the factors and the rectangles represent the items of the scale. ***p < 0.001; **p < * <0.005. The variance in each factor is defined in 1 by JASP^42^. All parameters were freely estimated in the analysis.

Initially, a CFA was conducted to assess the structural validity of the adapted instrument. The CFA results demonstrated acceptable fit indices, presenting a CFI of 0.991, GFI of 0.986, TLI of 0.989, RMSEA of 0.061, and SRMR of 0.061. Additionally, all items exhibited factor loadings above 0.40, indicating satisfactory internal structure validity. Table 1 present the factor loadings and Fig. 2 present the path model with the factors correlations.

**Table 1:** Factors loadings.

D	I	SE	Z-value	CI		$\lambda$
				5%	95%	
GQACC	GQACC1	0.021	40.108	0.810	0.894	**0.852**
	GQACC2	0.023	35.673	0.768	0.857	**0.813**
	GQACC3	0.027	27.783	0.698	0.804	**0.751**
	GQACC4	0.022	39.179	0.808	0.893	**0.850**
	GQACC5	0.023	35.272	0.771	0.862	**0.816**
	GQACC6	0.019	45.626	0.819	0.893	**0.856**
	GQACC7	0.032	20.296	0.586	0.711	**0.649**
	GQACC8	0.020	43.673	0.823	0.900	**0.862**
GQCH	GQCH1	0.025	31.040	0.726	0.824	**0.775**
	GQCH2	0.023	35.545	0.769	0.859	**0.814**
	GQCH3	0.028	25.781	0.678	0.789	**0.733**
	GQCH4	0.028	27.296	0.709	0.818	**0.764**
	GQCH5	0.035	17.751	0.556	0.694	**0.625**
	GQCH6	0.021	39.033	0.790	0.874	**0.832**
	GQCH7	0.021	42.676	0.841	0.921	**0.881**
	GQCH8	0.027	28.615	0.714	0.819	**0.767**
GQCP	GQCP1	0.016	52.872	0.838	0.902	**0.870**
	GQCP2	0.015	59.537	0.854	0.912	**0.883**
	GQCP3	0.018	49.242	0.848	0.918	**0.883**
	GQCP4	0.025	31.133	0.725	0.822	**0.773**
	GQCP5	0.027	30.759	0.769	0.874	**0.822**
	GQCP6	0.029	26.284	0.693	0.805	**0.749**
	GQCP7	0.029	26.811	0.718	0.831	**0.775**
GQGD	GQGD1	0.016	54.630	0.826	0.888	**0.857**
	GQGD2	0.017	47.339	0.792	0.860	**0.826**
	GQGD3	0.015	58.296	0.848	0.907	**0.878**
	GQGD4	0.023	33.848	0.728	0.817	**0.773**
	GQGD5	0.019	43.324	0.780	0.854	**0.817**
	GQGD6	0.022	39.497	0.814	0.899	**0.856**
	GQGD7	0.026	30.798	0.742	0.843	**0.793**
GQIM	GQIM1	0.036	19.557	0.633	0.773	**0.703**
	GQIM2	0.029	28.245	0.753	0.865	**0.809**
	GQIM3	0.025	30.926	0.729	0.828	**0.779**
	GQIM4	0.028	26.332	0.671	0.779	**0.725**
	GQIM5	0.046	9.887	0.362	0.540	0.451
	GQIM6	0.027	29.192	0.735	0.841	**0.788**
	GQIM7	0.023	35.423	0.770	0.860	**0.815**
	GQIM8	0.024	33.695	0.757	0.850	**0.803**
	GQIM9	0.024	33.581	0.771	0.867	**0.819**
GQPF	GQPF1	0.030	23.321	0.638	0.755	**0.697**
	GQPF2	0.028	26.168	0.668	0.777	**0.723**
	GQPF3	0.024	31.242	0.716	0.812	**0.764**
	GQPF4	0.031	21.527	0.609	0.731	**0.670**
	GQPF5	0.018	47.351	0.813	0.884	**0.849**
	GQPF6	0.027	26.909	0.676	0.782	**0.729**
	GQPF7	0.021	38.512	0.784	0.868	**0.826**
	GQPF8	0.022	35.562	0.748	0.836	**0.792**
	GQPF9	0.019	43.545	0.792	0.867	**0.830**
GQSE	GQSE1	0.014	62.762	0.851	0.906	**0.879**
	GQSE2	0.014	63.543	0.850	0.904	**0.877**
	GQSE3	0.013	67.415	0.876	0.928	**0.902**
	GQSE4	0.014	62.423	0.846	0.901	**0.874**
	GQSE5	0.016	52.156	0.814	0.878	**0.846**
	GQSE6	0.019	42.393	0.776	0.851	**0.813**
	GQSE7	0.015	55.630	0.819	0.879	**0.849**
	GQSE8	0.020	40.496	0.773	0.852	**0.812**

*N* = 384. D: Dimensions/factors; I: Items; SE: standard errors; CI: Confidence interval; $\lambda$ : standardized $\lambda$ ; bold: $\lambda$ $\ge$ 0.500; GQACC: Accomplishment; GQCH: Challenge; GQCP: Competition; GQGD: Guided; GQIM: Immersion; GQPF: Playfulness; GQSE: Social Experience.

### Internal reliability, correlations, and dimensions distribution

We analyzed the distributions of the responses for all variables by using the Shapiro-Wilk test^43^, a well-established method for normality assessment that is particularly suitable for samples of this size^53^. The results of the test yielded a W statistic of 0.937 for the accomplishment dimension, 0.954 for the challenge dimension, 0.955 for the competition dimension, 0.959 for the guided dimension, 0.983 for the immersion dimension, 0.979 for the playfulness dimension and 0.963 for the social experience dimension, and a *p-value* of < 0.001 for both dimensions, leading us to reject the null hypothesis of normality and conclude that the data exhibited a non-normal distribution, answering the dimension distribution test. We also measured the descriptive statistics (Mean, the standard deviation, and the data variances in each sub-questionnaire), the internal reliability analyses (Cronbach’s $\alpha$ and McDonald’s $\omega$), and the factor correlation coefficients, to discover if there is a relationship between two variables, and how strong that relationship may be^54^. Each GAMEFULQUEST sub-questionnaire has between 7 and 9 items, rated on a 7-point Likert scale. That way, the minimum value a sub-questionnaire can be is 7 and the maximum value a sub-questionnaire can be is 63. The internal reliability of each dimension of the adapted instrument was assessed using Cronbach’s $\alpha$ and McDonalds $\omega$ coefficients. The results (presented in the Table 2) indicate high levels of internal consistency across all dimensions.

**Table 2:** Internal reliability of each dimension.

Dimension	$\alpha$	$\omega$	M	Var	SD
Accomplishment	0.924	0.924	42.68	101.272	1.063
Challenge	0.908	0.908	39.4	108.344	10.409
Competition	0.911	0.911	33.67	112.44	10.604
Guided	0.923	0.924	32.57	101.505	10.075
Immersion	0.901	0.903	33.1	164.447	12.824
Playfulness	0.908	0.908	39.61	155.799	12.482
Social experience	0.944	0.944	28.07	164.568	12.828

*N* = 384. $\alpha$: Cronbachâ€™s $\alpha$; $\omega$: McDonald’s $\omega$; M: mean; Var: Variance; SD: Standard deviation.

These findings suggest that the items within each dimension of the instrument are highly correlated with each other^55^, indicating strong internal consistency (i.e., $\alpha \ge 0.600$). In the same way, the mean, variance, and standard deviation also maintain a balance in their values in each dimension, with a slight emphasis on the accomplishment dimension, which presented a significantly lower standard deviation and an average slightly above the others. On the other hand, the social experience dimension presented the lowest average of all.

The correlation between the dimensions of the adapted instrument were examined in the CFA process, and the results presented in the Fig. 2 revealed significant correlations between all dimensions. These results indicate that the dimensions of the instrument are related to each other, suggesting internal structure validity. However, following again Cohen^55^ classification table, which indicates that a strong correlation must present values above 0.50, moderate correlations present values between 0.30 and 0.50, and weak correlations present values between 0.10 and 0.30, the correlation values presented would be classified as, for the most part, between moderate and weak, except for the correlation between Accomplishment and Challenge, considered a strong correlation.

Finally, the coefficient of determination for each correlation was also calculated, which indicates how much one variable is associated with the other in terms of shared variance. The distribution of scores within each dimension of the adapted instrument was also examined. Although all the dimensions displayed non-normal distributions, they exhibited a range of scores that adequately captured the variability in participants’ responses.

### Summary of the results

Overall, the results suggest that the adapted instrument maintains good internal reliability, with high levels of internal consistency observed across all dimensions. Significant correlations between dimensions indicate internal structure validity, corroborating with the CFA results, that support the same aspect of the instrument. These findings provide confidence in the reliability and validity of the adapted instrument for measuring users’ gameful experience within gameful environments. Table 3 present the consolidated GAMEFULQUEST in English and adapted in Brazilian Portuguese. The questionnaire should be presented as follows (on a 7-point Likert scale):

**In English**: “Please indicate how much you agree with the following statements, regarding your feelings while using the chosen platform. Overall, chosen platform...”

**In Brazilian Portuguese**: “*Por favor, indique o quanto você concorda com as seguintes afirmações, sobre seus sentimentos ao usar a plataforma escolhida. No geral, a plataforma escolhida...*”

**Table 3:** Cross-cultural adapted instrument.

Original items	Adapted items (in Brazilian Portuguese)
**Accomplishment**	***Conquista***
Makes me feel that I need to complete things	*Me faz sentir a necessidade de completar as tarefas*
Pushes me to strive for accomplishments	*Me motiva a lutar por conquistas*
Inspires me to maintain my standards of performance	*Me inspira a manter meus padrões de desempenho*
Makes me feel that success comes through accomplishments	*Me faz sentir que o sucesso vem por meio das conquistas*
Makes me strive to take myself to the next level	*Faz com que eu me esforce para chegar ao próximo nível*
Motivates me to progress and get better	*Me motiva a progredir e me tornar melhor*
Makes me feel like I have clear goals	*Me faz sentir que eu tenho objetivos claros*
Gives me the feeling that I need to reach goals	*Me dá a sensação de que eu preciso alcançar os objetivos*
**Challenge**	***Desafio***
Makes me push my limits	*Me faz ir além dos meus limites*
Drives me in a good way to the brink of wanting to give up	*Me conduz, de um jeito bom, até meu limite*
Pressures me in a positive way by its high demands	*Me pressiona positivamente devido às suas altas exigências*
Challenges me	*Me desafia*
Calls for a lot of effort in order for me to be successful	*Exige muito esforço para que eu possa ser bem sucedido*
Motivates me to do things that feel highly demanding	*Me motiva a fazer as tarefas mais difíceis*
Makes me feel like I continuously need to improve in order to do well	*Me faz sentir que preciso continuar melhorando para me sair bem*
Makes me work at a level close to what I am capable of	*Me faz trabalhar a um nível próximo do que sou capaz*
**Competition**	**Competição**
Feels like participating in a competition	Me faz sentir como se estivesse em uma competição
Inspires me to compete	Me inspira a competir
Involves me with its competitive aspects	Me envolve por seus aspectos competitivos
Makes me want to be in first place	Me faz querer estar em primeiro lugar
Makes victory feel important	Me faz sentir que a vitória é importante
Feels like being in a race	Me faz sentir como se estivesse em uma corrida
Makes me feel that I need to win to succeed	Me faz sentir que preciso vencer para ter sucesso
**Guidance**	***Condução***
Makes me feel guided	*Me faz sentir guiado*
Gives me a sense of being directed	*Me dá a sensação de estar sendo direcionado*
Makes me feel like someone is keeping me on track	*Me faz sentir como se alguém estivesse me mantendo no caminho certo*
Gives me the feeling that I have an instructor	*Me dá a sensação de que tenho um instrutor*
Gives me the sense I am getting help to be structured	*Me dá a sensação de que estou recebendo ajuda para me organizar*
Gives me a sense of knowing what I need to do to do better	*Me dá a sensação de saber o que eu preciso fazer para melhorar*
Gives me useful feedback so I can adapt	*Me dá um retorno útil para que eu possa me adaptar*
**Immersion**	***Imersão***
Gives me the feeling that time passes quickly	*Me dá a sensação de que o tempo está passando rápido*
Grabs all of my attention	*Chama minha atenção por completo*
Gives me a sense of being separated from the real world	*Me dá a sensação de estar fora do mundo real*
Makes me lose myself in what I am doing	*Me faz perder a noção de mim mesmo naquilo que estou fazendo*
Makes my actions seem to come automatically	*Faz com que minhas ações pareçam ser automáticas*
Causes me to stop noticing when I get tired	*Faz com que eu pare de perceber quando fico cansado*
Causes me to forget about my everyday concerns	*Faz com que eu esqueça minhas preocupações cotidianas*
Makes me ignore everything around me	*Me faz ignorar tudo ao meu redor*
Gets me fully emotionally involved	*Me deixa totalmente envolvido emocionalmente*
**Playfulness**	***Ludicidade***
Gives me an overall playful experience	*Me dá uma experiência lúdica geral*
Leaves room for me to be spontaneous	*Deixa espaço para eu ser espontâneo*
Taps into my imagination	*Estimula minha imaginação*
Makes me feel that I can be creative	*Me faz sentir que posso ser criativo*
Gives me the feeling that I explore things	*Me dá a sensação de que exploro as tarefas*
Feels like a mystery to reveal	*Parece um mistério a ser revelado*
Gives me a feeling that I want to know what comes next	*Me dá a sensação de que quero saber o que vem a seguir*
Makes me feel like I discover new things	*Me faz sentir como se descobrisse coisas novas*
Appeals to my curiosity	*Estimula a minha curiosidade*
**Social experience**	***Experiência Social***
Gives me the feeling that Iâ€™m not on my own	*Me dá a sensação de que não estou sozinho*
Gives me a sense of social support	*Me dá a sensação de apoio social*
Makes me feel like I am socially involved	*Me faz sentir socialmente envolvido*
Gives me a feeling of being connected to others	*Me dá a sensação de estar conectado a outros*
Feels like a social experience	*Parece uma experiência social*
Gives me a sense of having someone to Share my endeavors with	*Me dá a sensação de ter alguém com quem compartilhar meus esforços*
Influences me through its social aspects	*Me influencia através de seus aspectos sociais*
Gives me a sense of being noticed for what I have achieved	*Me dá a sensação de estar sendo notado por aquilo que conquistei*

## Discussion

In this study, we conducted a cross-cultural adaptation of the GAMEFULQUEST Questionnaire proposed by Högberg, Hamari, and Wästlund^1^, followed by an analysis of its psychometric properties. The adaptation process involved six steps outlined by Borsa, Damásio, and Bandeira^30^, including translation, synthesis, expert evaluation, target audience assessment, adapted instrument application, and validation. The results obtained in all phases of the cross-cultural analysis are presented in the final adapted instrument.

The results showed that there is a correlation between all items, especially when we observe the internal correlation of the dimension sub-questionnaires. When it comes to correlations between dimensions, the most notable was between the achievement and challenge dimensions. The CFA presented a good model fit ($\chi ^2/df$ = 2.4, RMSEA = 0.061, CFI = 0.991, TLI = 0.989, GFI = 0.986 and SRMR = 0.061), numbers that are within the recommended parameters, with only one variation in the RMSEA index, which reached 0.061, a value 0.001 above the maximum margin, considering the parameters indicated by Hu & Bentler, which stipulate a cutoff limit close to 0.06 for the index^50^. However, the fit indices generally demonstrate success in the model, which demonstrates that the adaptation was carried out satisfactorily.

In a direct comparison with the original instrument, we can identify significant points of convergence. The adapted questionnaire achieved higher values in all indices, such as CFI (0.928 in the original, against 0.991 in the adaptation), TLI (0.924 in the original, against 0.989 in the adaptation), RMSEA (0.046 in the original, against 0.061 in the adaptation) and SRMR (0.0561 in the original, against 0.061 in the adaptation), as well as maintaining its factor loadings on the items always above 0.4. Likewise, Cronbach’s alpha remained above 0.9 in all dimensions, surpassing the average of 0.7 of the original instrument. Likewise, McDonald’s $\omega$ remained above 0.9 in all dimensions, including values mostly identical to those presented by Cronbach’s alpha, except for the dimensions Guided, where it presented a value of 0.001 above, and Immersion, presenting a value of 0.002 above. However, the correlation values were lower than those of the original instrument, when referring to the correlation between dimensions, since the correlation between items of the same dimension proved to be quite strong.

Some interesting points could be observed individually in the steps carried out. For example, in the translation synthesis stage, we sought to maintain a balanced language, preserving the academic character of the instrument, but with expressions and syntactic constructions closer to colloquial language, more easily accepted by audiences of all contexts, social levels, and knowledge. Some specific items presented greater complexity in their translation, for example, item 2 of the challenge sub-questionnaire, which read in the original version “Drives me in a good way to the brink of wanting to give up”. The term “brink”, when translated literally, becomes “beira”, a term that has proven problematic for some people to understand. The construction of the item as a whole, with the opposition of ideas (being taken positively next to a negative attitude), also caused strangeness, which led to a slightly deeper adaptation, which can also be seen in the following stages, culminating in a considerably less complex final version. Other items presented this phenomenon, such as the title of the guided sub-questionnaire itself, which needed to be adapted to a more accepted term in Portuguese (driving). However, surprisingly, when used in item one “Makes me feel guided” of the dimension, the term managed to be maintained with its literal translation “guiado”, because in the context of the item, it was understood. A similar phenomenon occurred with terms such as “taps” (item three of the playfulness sub-questionnaire), which was understood in different ways by the translators (“Stimulates” and “Explores”), or “Appeals” (item nine of the playfulness questionnaire), where the term “Apela” in Portuguese is not commonly used by the lay population in general.

Subsequently, with the expert and target audience evaluation stages, an even more detailed refinement of the construction of the items can be observed. It was possible to observe, in the experts’ stage, a concern with the construction of specific items, so that these, even with a more simplified language, maintained their psychometric properties of measuring the specific points of the construct. An example can be seen in item two of the achievement sub-questionnaire (Pushes me to strive for accomplishments). When translated and synthesized, its composition was changed to “Me motivates me to seek achievements”. However, as pointed out by the experts, the expression “strive for”, in a general context, would be better adapted to “fight for”, a suggestion that was accepted and changed.

Similarly, when presenting the instrument for analysis of the target audience, there were several suggestions for simplifying the language, bringing the items closer to colloquial language. However, care was taken when making changes, in order not to mischaracterize the instrument and its properties. A notable change suggested was the replacement of the term “things” (things, in the original versions), which needed to be replaced by something “more concrete”, in the words of some evaluators.

Overall, after all the analyses conducted in this study, the results demonstrated that the Brazilian Portuguese version of the GAMEFULQUEST is an instrument that is near complete validation. The questionnaire evaluated in this study can be used to measure the gameful experience of users in playful platforms, in future research involving Brazilian samples. The use of this translated instrument can be an effective option for researchers and practitioners to evaluate the impacts of the platforms, based on the reports of the gameful feelings, as well as provide information to personalize gameful environments or conduct new analysis about factors that can be changed, to improve the results of the application of platforms.

## Limitations and opportunities for the future

This study delineates certain limitations that warrant consideration. Regarding the demographic data of respondents, we encountered challenges in securing responses from all Brazilian states, with certain regions experiencing low participation. This limitation hinders our ability to elucidate the potential use of the instrument considering the linguistic variations of the country. Moreover, the age distribution of respondents skewed towards individuals within a certain age limit, thereby limiting the generalizability of the results to children, teenagers, and the elderly.

Another point of limitation found was the inability to maintain more detailed control over the interaction of participants with the indicated platform (in the case of the first form) or chosen (second form). Despite the guidance to answer the questionnaire only after at least 20 min of using the platform, it proved impossible to effectively monitor whether this rule was followed to the letter. Finally, it was not possible to perform the gender invariance analysis, since the sample size achieved did not meet the minimum quantity to perform such a test efficiently and reliably. While we scrutinized the psychometric properties of the GAMEFULQUEST Questionnaire translated into Brazilian Portuguese, it is important to note that other countries with Portuguese as the official language (e.g., Portugal, Angola, Mozambique) may find the instrument used in this study unsuitable for their contexts.

In light of these limitations, we propose avenues for future research. Firstly, we recommend studies specifically scrutinizing the psychometric properties of the Brazilian Portuguese questionnaire for children and teenagers (especially considering that this audience tends to consume gameful environments). This approach aligns with previous endeavors that sought to validate the questionnaire for younger age groups. Such validation efforts with adolescents can offer valuable insights for designers seeking to tailor gameful environments.

Secondly, Brazil is a vast country, with different regions and states having their linguistic variations. Therefore, conducting cross-cultural studies may not encompass all the linguistic variations present. To address this issue, we recommend the realization of new studies in all the regions of the country, to cover as many regional variations as possible. We recommend, also, the development of control mechanisms for how respondents use the chosen platform so that there can be a more detailed standardization of the situations faced individually when answering the instrument, generating even more consistent results. Lastly, recognizing the cultural and linguistic differences among countries where Portuguese is the official language, future studies should undertake the adaptation of the Brazilian Portuguese questionnaire for use in other Portuguese-speaking nations, thereby enabling its broader applicability.

## Conclusion

Overcoming the language barrier, and providing researchers with reliable instruments for measuring the most diverse constructs, is a challenge. This study successfully conducted a cross-cultural adaptation of the GAMEFULQUEST questionnaire and examined its psychometric properties in the Brazilian context. The instrument was carefully adapted, incorporating feedback from experts and the target audience to ensure its validity and reliability. The CFA provided evidence of the structural validity of the adapted instrument, while measures of internal reliability and correlations between dimensions supported its internal consistency and internal structure validity, respectively. The availability of a validated instrument for measuring gameful experience in the Brazilian context, not only facilitates research in the field of gamification but also opens possibilities for the design and evaluation of gameful interventions, tailored to the needs and preferences of Brazilian users. In future research, we aim to explore the applicability of the adapted instrument across different cultural contexts (i.e., demographic region, gender, and age), since Brazil is a country of continental dimensions, with countless different realities.

## Data Availability

All data generated or analyzed during this study are included in this published article and its supplementary information files.
