# Author Correction: Adaptation and psychometric investigation of the Gameful Experience Questionnaire (GAMEFULQUEST) in Brazilian Portuguese

**DOI:** 10.1038/s41598-025-02761-x

**Published:** 2025-05-22

**Authors:** Luiz Oliveira da Silva Junior, Wilk Oliveira, Juho Hamari

**Affiliations:** 1 Federal University of Paraíba, Rio Tinto, Brazil; 2 Gamification Group, Faculty of Information Technology and Communication Sciences, Tampere University, Tampere, Finland

Correction to: *Scientific Reports* 10.1038/s41598-024-68101-7, published online 26 July 2024

In the original version of this Article, the items related to the ‘Competition’ dimension were missing from Table 3.

As a result, Table 3 now reads:

**Table:** 

Original items	Adapted items (in Brazilian Portuguese)
**Competition**	***Competição***
Feels like participating in a competition	*Me faz sentir como se estivesse em uma competição*
Inspires me to compete	*Me inspira a competir*
Involves me with its competitive aspects	*Me envolve por seus aspectos competitivos*
Makes me want to be in first place	*Me faz querer estar em primeiro lugar*
Makes victory feel important	*Me faz sentir que a vitória é importante*
Feels like being in a race	*Me faz sentir como se estivesse em uma corrida*
Makes me feel that I need to win to succeed	*Me faz sentir que preciso vencer para ter sucesso*

The original Article has been corrected.

